# Anaerobic biosynthesis of rhamnolipids by *Pseudomonas aeruginosa*: performance, mechanism and its application potential for enhanced oil recovery

**DOI:** 10.1186/s12934-021-01593-4

**Published:** 2021-05-20

**Authors:** Feng Zhao, Qingzhi Wang, Ying Zhang, Liying Lei

**Affiliations:** 1 School of Life Sciences, Qufu Normal University, Qufu, 273165 Shandong Province China; 2 Institute of Applied Ecology, Chinese Academy of Sciences, Shenyang, 110016 Liaoning Province China

**Keywords:** *Pseudomonas aeruginosa*, Rhamnolipids, Anaerobic biosynthesis, Glycerol, *RmlBDAC*, Microbial enhanced oil recovery

## Abstract

**Background:**

*Pseudomonas aeruginosa*, the rhamnolipids-producer, is one of dominant bacteria in oil reservoirs. Although *P. aeruginosa* strains are facultative bacteria, the anaerobic biosynthesis mechanism of rhamnolipids is unclear. Considering the oxygen scarcity within oil reservoirs, revealing the anaerobic biosynthesis mechanism of rhamnolipids are significant for improving the in-situ production of rhamnolipids in oil reservoirs to enhance oil recovery.

**Results:**

*Pseudomonas* *aeruginosa* SG anaerobically produced rhamnolipids using glycerol rather than glucose as carbon sources. Two possible hypotheses on anaerobic biosynthesis of rhamnolipids were proposed, the new anaerobic biosynthetic pathway (hypothesis 1) and the highly anaerobic expression of key genes (hypothesis 2). Knockout strain SGrmlB failed to anaerobically produce rhamnolipids using glycerol. Comparative transcriptomics analysis results revealed that glucose inhibited the anaerobic expression of genes *rmlBDAC*, *fabABG*, *rhlABRI*, *rhlC* and *lasI*. Using glycerol as carbon source, the anaerobic expression of key genes in *P. aeruginosa* SG was significantly up-regulated. The anaerobic biosynthetic pathway of rhamnolipids in *P. aeruginosa* SG were confirmed, involving the gluconeogenesis from glycerol, the biosynthesis of dTDP-l-rhamnose and -hydroxy fatty acids, and the rhamnosyl transfer process. The engineered strain *P. aeruginosa* PrhlAB constructed in previous work enhanced 9.67% of oil recovery higher than the wild-type strain *P. aeruginosa* SG enhancing 8.33% of oil recovery.

**Conclusion:**

The highly anaerobic expression of key genes enables *P. aeruginosa* SG to anaerobically biosynthesize rhamnolipids. The genes, *rmlBDAC*, *fabABG*, *rhlABRI*, *rhlC* and *lasI*, are key genes for anaerobic biosynthesis of rhamnolipid by *P. aeruginosa*. Improving the anaerobic production of rhamnolipids better enhanced oil recovery in core flooding test. This study fills the gaps in the anaerobic biosynthesis mechanism of rhamnolipids. Results are significant for the metabolic engineering of *P. aeruginosa* to enhance anaerobic production of rhamnolipids.

## Background

At present, the demand for oil in economic development continues to increase, while the recoverable oil reserves in oil reservoirs are decreasing, and it is more difficult to find new oil and gas resources. It is of great strategic significance to enhance the oil recovery of existing oil fields through scientific research and innovation. Microbial oil recovery technology (MEOR) is an economical, effective and environmentally friendly oil recovery technology [1]. It is feasible to enhance oil recovery using microorganisms to in-situ produce biosurfactants in oil reservoirs [2,4]. It is also proved that the stable microbial growth and efficient production of biosurfactants such as rhamnolipids in reservoirs are the keys to the successful implementation of MEOR [1, 3, 4]. Rhamnolipids can effectively reduce the oilwater interfacial tension, emulsify crude oil and change the wettability of reservoir rock, so it is one of the excellent oil displacement agents used to improve oil recovery [1, 4]. The environments in oil reservoirs are anoxic or anaerobic [5]. The injection of air into oil reservoirs is difficult to ensure the effective oxygen supply of aerobic microorganisms [4]. However, studies on anaerobic synthesis of biosurfactants are still weak [6], which limits to enhance oil recovery by anaerobic production of biosurfactants in oil reservoirs.

Although rhamnolipids as a natural biosurfactants is extensively studied and applied in MEOR and other fields [7, 8], studies on anaerobic biosynthesis of rhamnolipids are also scarce [6]. Many rhamnolipid-producing strains have been repoted, such as *Pseudomonas* sp. and *Burkholderia* sp. [8]. Among the rhamnolipid-producing strains, *Pseudomonas aeruginosa* has the highest rhamnolipid yield [8]. Therefore, *P. aeruginosa* was mainly studied for producing rhamnolipids [8, 9]. *P. aeruginosa* are facultative bacterial strains that can grow and metabolize at both aerobic and anaerobic conditions [6, 10, 11]. But most of the studies on rhamnolipids production by *P. aeruginosa* were focused on aerobic biosynthesis.

Studies have discovered that some *P. aeruginosa* strains can anaerobically produce rhamnolipids when using glycerol as carbon source [12,15]. But the related anaerobic biosynthesis mechanisms of rhamnolipids are unclear. How to further enhance the anaerobic yield of rhamnolipids requires the modification of key genes or the regulation of the anaerobic biosynthetic pathways, which all depend on the anaerobic biosynthesis mechanisms of rhamnolipids.

This study aims to explore the underlying anaerobic biosynthetic mechanism of rhamnolipids when *P. aeruginosa* SG using glycerol as carbon source. Strain *P. aeruginosa* SG was used for rhamnolipids production [13, 14]. *P. aeruginosa* SG was cultured with different carbon sources (glucose and glycerol). Cell growth and rhamnolipids production were comparatively studied. Two possible hypotheses on anaerobic biosynthesis of rhamnolipid using glycerol were proposed. RNA sequencing (RNA-seq) was performed on the anaerobic culture of strain SG using glucose and glycerol as carbon source, respectively. The transcriptomic data were analyzed for the anaerobic biosynthesis pathways of rhamnolipids and related genes regulation. The method of gene knock-out was used to identify the key genes and the anaerobic biosynthetic pathway of rhamnolipids when *P. aeruginosa* SG using glycerol. Application potential by anaerobic production of rhamnolipids for MEOR was evaluated and discussed. Results will help to regulate *P. aeruginosa* to produce more rhamnolipids under anaerobic conditions. This study also provides scientific guidance for the development of MEOR technology through in situ production of rhamnolipids.

## Materials and methods

### Bacterial strains and culture conditions

Strain *P*.* aeruginosa* SG was used as a rhamnolipids-producer in this study. *P. aeruginosa* SG can anaerobically produce rhamnolipids [13, 14]. *P. aeruginosa* SG was cultured in LB (LuriaBertani) medium to prepare seed culture at 35C and 180rpm for 12h. To avoid interference of rhamnolipids produced in seed culture, the seed culture was centrifuged at 5000*g* for 2min. The bacterial cells were washed twice using sterile distilled water. The suspension liquid of bacterial cells with sterile distilled water was transferred into the fermentation medium. The fermentation medium except for carbon source contained 4g/l of NaNO_3_, 3g/l of KH_2_PO_4_, 4g/l of K_2_HPO_4_3H_2_O, 0.5g/l of KCl, 0.5g/l of NaCl, 0.2g/l of CaCl_2_2H_2_O, 1.0g/l of MgSO_4_7H_2_O. Glucose and glycerol were used as carbon sources with the concentrations of 40g/l. The aerobic culture was performed in 250ml-triangular flasks containing 100ml fermentation medium, at 35C and 180rpm for 6days. The anaerobic culture was performed in 100ml-serum bottles containing 80ml anaerobic fermentation medium. The anaerobic medium was prepared as described in previous studies [13, 16]. The anaerobic culture was incubated at 35C and 50rpm for 8days. Three parallels were set for each experiments. The inoculum amount was 3% (v/v). The non-inoculated medium was used as the negative control. *Escherichia coli* DH5, *E. coli* S17-1 and recombinant strains were cultured in LB medium at 35C and 180rpm. During gene knock-out process, LB medium containing ampicillin of 100mg/l and kanamycin of 50mg/l were used for recombinant *E. coli* strains. LB medium containing kanamycin of 350mg/l was used for recombinant *P. aeruginosa* strains.

### Analytical methods

The anaerobic culture was sampled from serum bottles using sterile syringes. Cell growth of strain SG was represented by OD_600_ values in the anaerobic culture. Then samples were centrifuged at 10,000*g* for 10min, respectively. The cell free supernatant was collected. The surfactants anaerobically produced by *P. aeruginosa* SG using glycerol were rhamnolipids which were confirmed by analytical methods of FTIR and HPLC-MS [14]. In this study, rhamnolipids concentration was also represented by the diameter of oil spreading circle formed by cell free supernatant. The diameter of oil spreading circle was measured as described in previous studies [17, 18]. Then residue supernatant surface tension was measured by surface tensiometer (BZY-1, Shanghai Hengping Instrument and Meter Factory, Shanghai, China).

### Gene *rmlB* knock-out

In this study, using the suicidal plasmid pK18mobSacB of *P. aeruginosa*, gene *rmlB* was knocked out to block the biosynthetic pathway of dTDP-L-rhamnose controlled by *rmlBDAC* operon genes. The *rmlBDAC* operon genes control the biosynthesis of dTDP-rhamnose [19]. The gene fragment rmlBD was obtained by PCR using the genomic DNA of *P. aeruginosa* SG as the template. The PCR primers were rmBD-f: 5-CGGAAGCTTATGTGGACCGCTCGAT-3 and rmBD-r: 5-GCCGAATTCCTGTTGCAGCTTGCGGT-3. The *rmlBD* fragment was 1969bp with restriction sites of *Hind* III and *EcoR* I at the 5 end and 3 end, respectively. The *rmlBD* fragment was cloned into pMD19T (simple) vector to construct plasmid pMD19-rmlBD. The *rmlBD* fragment contains two *Eco52* I restriction sites at 831bp and 1206bp. After pMD19T-rmlBD successively digested with *Eco52* I enzyme and T4 DNA ligase, plasmid pMD19-rmlBD was constructed and purified by gel extraction kit (Takara, Japan). The rmlBD fragment was cloned into the *Hind* III and *EcoR* I sites of plasmid pK18mobSacB to construct recombinant plasmid pK18-rmlBD. The recombinant plasmid pK18-rmlBD was transformed into strain *E. coli* S17-1. Using conjugation method [20], the plasmids pK18-rmlBD were transferred into the strain *P. aeruginosa* SG. Mutants were cultured on LB medium containing Ampicillin and kanamycin for the first recombination. The LB medium containing 20% sucrose and Ampicillin was used to pick out the knockout strain *P*.* aeruginosa* SGrmlB. The anaerobic cell growth and anaerobic rhamnolipid production of wild-type strain SG and the knockout strain SGrmlB were comparatively determined.

### RNA extraction, library construction and RNA sequencing

*P*.* aeriginosa* SG cells were sampled from its anaerobic culture with OD_600_=0.6. *P*.* aeriginosa* SG cultured with glucose and glycerol as carbon sources were marked as GluAn and GlyAn, respectively. Three replicates, GluAn-1, GluAn-2, GluAn-3 and GlyAn-1, GlyAn-2, GlyAn-3, were sampled for GluAn and GlyAn. The bacterial cells were collected by centrifugation (4C, 4000*g*, 5min). The harvested bacterial cells were used for the total RNA extraction. The Takara RNA kit (Takara, Japan) was used for RNA extraction and purification according to the manufacturer provided protocol. Then RNA purity of GluAn samples and GlyAn samples was evaluated by Nanodrop2000 with OD_260/280_ values and OD_260/230_ values. RNA fragment length of GluAn samples and GlyAn samples was determined using Agilent 2100 Bioanalyzer. The tested mRNA was enriched by removing rRNA. After enrichment, mRNA is fragmented into short fragments. First strand of cDNA was synthesized by reverse transcription using random hexamers and mRNA as template, and then the buffer, dNTPs and DNA polymerase I were added to synthesize the two-strand cDNA. Then AMPure XP Beads were used to purify double-stranded cDNA. The purified double stranded cDNA was treated with terminal repair, addition of A and joint. The cDNA libraries were constructed using the method of chain-specific library by the Allwegene BioTech in Beijing (China). RNA sequencing was performed using Illumina Hiseq 4000 with PE150 double terminal sequencing strategy by the Allwegene BioTech in Beijing (China).

### Transcriptome analysis

The obtained raw reads were filtered to remove the reads with sequencing adapter and the low-quality Reads. The sequencing data were statistically analyzed for raw reads numbers, clean reads numbers, sequence error rate, Q20 and Q30 (proportion of clean data with Phred value greater than 20 and 30) and GC content (%). The high quality data, clean reads, were used for transcriptome analysis [21]. The obtained clean reads were mapped to the reference genome of *P. aeruginosa* PAO1 using Bowtie2 alignment software. The regional distribution and Reads density distribution were evaluated. The gene annotation, gene structure analysis and the new transcript prediction were performed.

FPKM (Fragments per kilobase of exon model per million mapped reads) values were calculated using HTSeq software [22] for the gene expression level analysis of each sample. The number of genes and the expression level of a single gene at different expression levels were counted respectively, and the FPKM value of one was used as the threshold to judge whether the gene was expressed or not. Using DESeq method [23], gene differential expression analysis was performed according to the readCount data obtained from gene expression level analysis. The differentially expressed genes between GluAn samples and GlyAn samples were identified with log2(FoldChange) value>1 and q-value<0.05. Hierarchical clustering analysis of differentially expressed genes was performed based on their expression levels, log10(FPKM+1). The GO (Gene Ontology, http://www.geneontology.org/) enrichment analysis was carried out using GOseq software [24], and the probability of GO term enriched by differential genes was calculated. The differentially expressed genes were enriched into molecular function, biological process, cellular component. KEGG (Kyoto Encyclopedia of Genes and Genomes, http://www.kegg.jp) enrichment analysis was performed for pathways enrichment of the differentially expressed genes [25].

### Pathways description and genes discovery

The metabolic pathways related to fatty acid metabolism, glycometabolism and rhamnosyl transfer process were mined from the transcripts information that has be enriched into KEGG pathways. Referring to the known information of aerobic biosynthetic pathways of rhamnolipid in model strain *P. aeruginosa* PAO1, the anaerobic biosynthetic pathways of rhamnolipid were attempted to analyze, starting with the metabolism of glycerol. Two precursors of rhamnolipid biosynthesis, dTDP-l-rhamnose and -hydroxy fatty acids, were of particular consideration. In the transcriptome of *P. aeruginosa* SG using glycerol under anaerobic conditions, the anaerobic biosynthesis of dTDP-l-rhamnose and -hydroxy fatty acids and the transfer process of rhamnosyl were the target biosynthetic pathways. When *P. aeruginosa* SG using glucose as carbon source under anaerobic conditions, the related biosynthetic pathways were also comparatively analyzed.

The transcriptome data of *P. aeruginosa* SG using glucose and glycerol as carbon source under anaerobic conditions were compared and analyzed. The related key genes that were significantly up-regulated or activated when using glycerol as carbon source were mined from the differentially expressed genes between GluAn samples and GlyAn samples. Referring to the known key genes of aerobic biosynthetic pathways of rhamnolipid in model strain *P. aeruginosa* PAO1, the candidate key genes involving in the anaerobic biosynthesis of rhamnolipid using glycerol were picked out from the significantly up-regulated genes. The genes related to the biosynthesis of dTDP-l-rhamnose and -hydroxy fatty acids, and the rhamnosyl transfer process were the target genes.

### Core flooding test

Enhanced oil recovery by in-situ anaerobic production of rhamnolipids was evaluated by core flooding test. The experimental protocol of core flooding test was referred to the previous study [13]. The engineered strain *P. aeruginosa* PrhlAB with higher anaerobic yield of rhamnolipids was used in core flooding tests. The engineered strain *P. aeruginosa* PrhlAB was constructed by increasing the copy number of *rhlAB* genes in *P. aeruginosa* SG [15]. The rock core has length of 293mm, diameter of 38mm, absolute permeability of 0.388m^2^, pore volume of 69.9ml, respectively. The Xinjiang oilfield production water and crude oil (density of 0.886g/cm^3^ and viscosity of 6.3mPas) were used. After the first water flooding, 0.5 PV of culture solution [strain PrhlAB and medium (1:20, v/v)] was injected into rock core. The medium contained 40g/l glycerol, 4g/l of NaNO_3_, 3g/l of KH_2_PO_4_, 4g/l of K_2_HPO_4_3H_2_O, 0.5g/l of KCl, 0.5g/l of NaCl, 0.2g/l of CaCl_2_2H_2_O. Then the rock core was incubated at 39C for 8days. During the core flooding process, the volumes of displaced oil (ml) and displaced water (ml) were recorded. Enhanced oil recovery efficiency (%) was calculated.

## Results and discussion

### Rhamnolipids production and cell growth using glucose and glycerol

Under aerobic conditions, strain *P. aeruginosa* SG decreased the culture surface tension to 27.0mN/m and 27.3mN/m using both glucose and glycerol as carbon sources, respectively. The oil spreading circles diameters of the aerobic culture were 482mm and 565mm when *P. aeruginosa* SG using glucose and glycerol as carbon sources, respectively. The surface activity and oil spreading activity indicate that *P. aeruginosa* SG produced biosurfactants [17, 18]. The produced biosurfactants by *P. aeruginosa* was rhamnolipids [14, 15]. Under aerobic conditions, *P. aeruginosa* SG produced rhamnolipids using both glucose and glycerol as carbon sources.

**Fig. 1:**
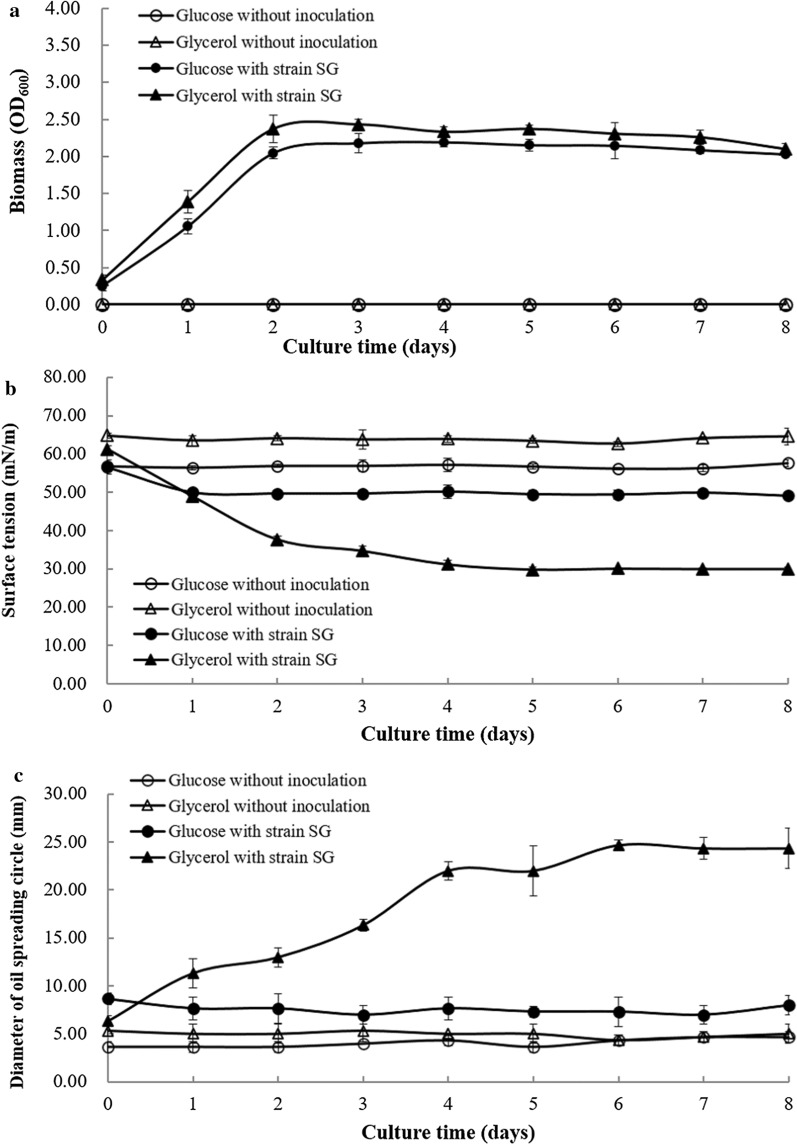
Anaerobic growth and production of rhamnolipids by *P. aeruginosa* SG using glucose and glycerol as carbon sources **a** biomass (OD_600_), **b** surface tension and **c** oil spreading activity

But rhamolipids production by strain SG under anaerobic conditions were quite different. Under anaerobic conditions, the cell growth (OD_600_), surface tension and oil spreading circles diameters were shown in Fig.1, From the biomass (OD_600_) point of view, strain *P. aeruginosa* SG grew well under anaerobic conditions using both glucose and glycerol as carbon sources (Fig.1a). However, the surface tension values of anaerobic culture were decreased from 64 to 30mN/m using glycerol (Fig.1b). While the surface tension values of anaerobic culture were decreased from 57 to 48mN/m using glucose. Results indicated that strain *P. aeruginosa* SG produced biosurfactants when using glycerol as carbon source. As shown in Fig.1c, the oil spreading circles diameters of anaerobic culture were 24mm and 8mm when strain *P. aeruginosa* SG using glucose or glycerol as carbon sources, respectively. The oil spreading activity also demonstrates that *P. aeruginosa* SG produced biosurfactants when using glycerol as carbon source [17, 18]. The anaerobically produced biosurfactants by *P. aeruginosa* SG was identified as rhamnolipids [13]. Results confirmed that *P. aeruginosa* SG can anaerobically grow well using both glucose and glycerol, but *P. aeruginosa* SG anaerobically produces rhamnolipids when using glycerol as carbon sources.

### Research hypotheses for anaerobic biosynthesis of rhamnolipids using glycerol

Under aerobic conditions, dTDP-rhamnose and -hydroxy fatty acids are two required precursors for rhamnolipids biosynthesis [19]. The gluconeogenesis pathway, the EntnerDoudoroff pathway and de novo synthesis of fatty acids are all central metabolic pathways in *P. aeruginosa*. These metabolic pathways can also support the growth of *P. aeruginosa* under anaerobic conditions [10, 26]. Therefore, under anaerobic conditions, glucose can be transformed into glucose-6-phosphate and then glucose-1-phosphate that was used for dTDP-l-rhamnose biosynthesis. Through catabolic pathways, both glucose and glycerol can link with the de novo synthesis pathway of fatty acids under anaerobic conditions. And then -hydroxy fatty acids can be synthesized by RhlA enzyme. Theoretically, the two precursors, dTDP-l-rhamnose and -hydroxy fatty acids, can be synthesize using both glucose and glycerol under anaerobic conditions. Therefore, rhamnolipids should be anaerobically synthesized by *P. aeruginosa* using both glycerol and glucose as carbon sources. In fact, *P. aeruginosa* anaerobically synthesized rhamnolipids mere using glycerol rather than glucose as carbon source.

**Fig. 2:**
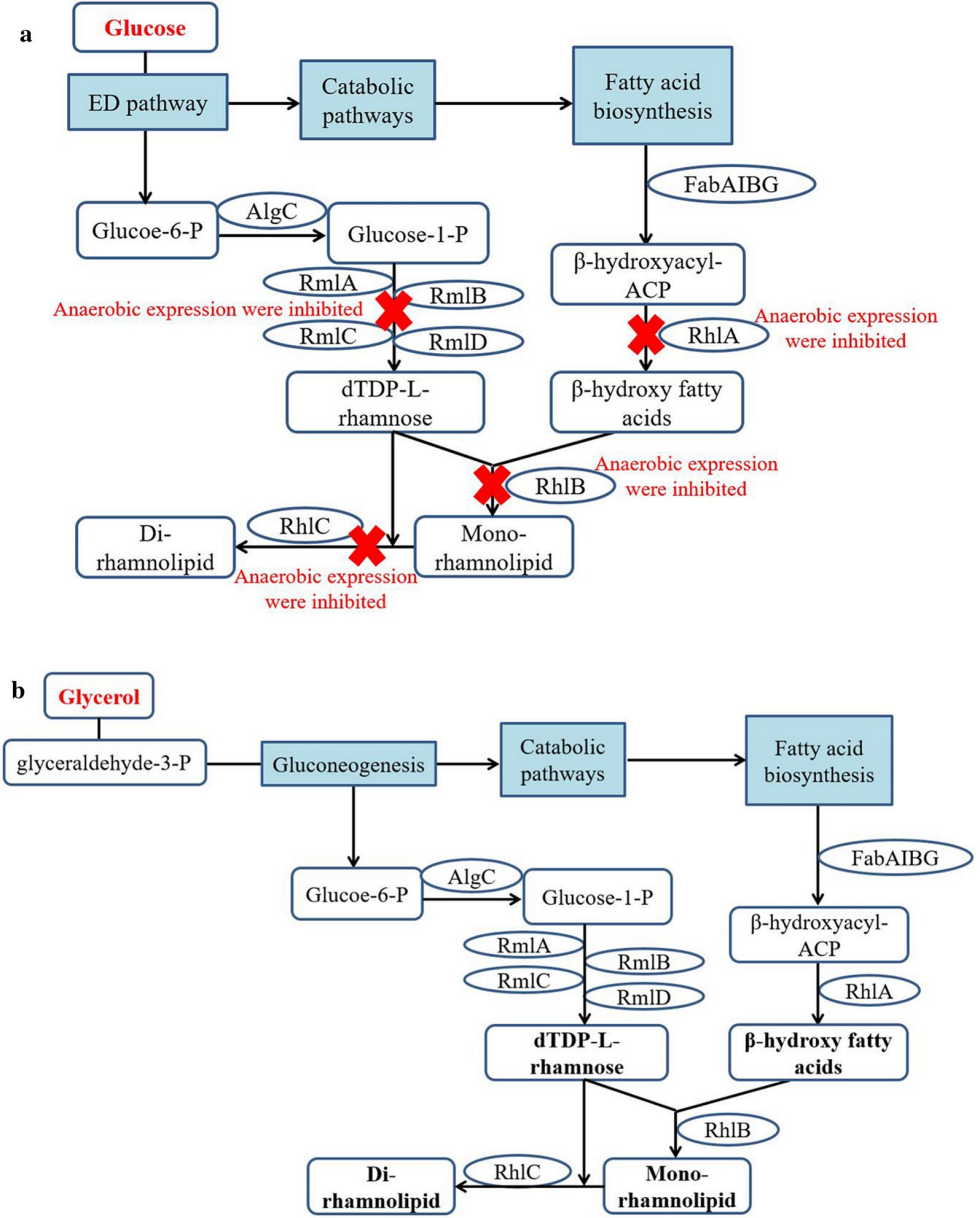
The anaerobic biosynthetic pathways of rhamnolipid in *P. aeruginosa* SG **a** using glucose as carbon source; **b** using glycerol as carbon source

Here, two possible hypotheses on anaerobic biosynthesis of rhamnolipid by *P. aeruginosa* using glycerol were proposed. Hypothesis 1: in *P. aeruginosa*, the anaerobic biosynthetic pathway of rhamnolipid is different from the aerobic biosynthetic pathway of rhamnolipids. This hypothesis suggests that anaerobic conditions block the biosynthesis of dTDP-l-rhamnose from glucose-1-phosphate, which results in *P. aeruginosa* being unable to anaerobically synthesize rhamnolipids from glucose. There may be other pathways to metabolize glycerol to synthesize dTDP-l-rhamnose without the effect of *rmlBDAC* operon genes, which leads to the anaerobic biosynthesis of rhamnolipids using glycerol. Hauser and Karnovsky (1957) cultured *P. aeruginosa* using ^14^C-labeled glycerol--^14^C and glycerol--^14^C as carbon sources. They found that the C_6_ skeleton of rhamnose was composed of two ^14^C-labeled C_3_ units that were derived from two molecules glycerol (^14^C-labeled C_3_ unit) without carbon chain rearrangement [27]. Hypothesis 2 was shown in Fig.2. In *P. aeruginosa*, the rhamnolipids biosynthetic pathway is same under both aerobic and anaerobic conditions, but the anaerobic expression of key genes for rhamnolipids synthesis is inhibited using glucose as carbon source, and these key genes can be highly expressed under anaerobic conditions using glycerol as carbon source. Previous studies compared the gene expression differences of *P. aeruginosa* using glucose as carbon source under aerobic and anaerobic conditions, and the results showed that the expressions of *rhlR*, *rhlI* and *rhlAB* genes related to rhamnolipids synthesis were inhibited under anaerobic conditions using glucose [28,30].

From the two hypotheses point of view, the anaerobic synthesis of dTDP-rhamnose is one key metabolic pathway for rhamnolipids biosynthesis under anaerobic conditions. Knocking out the *rmlBDAC* operon can identify whether the *rmlBDAC* genes involved in the anaerobic biosynthesis of rhamnolipid using glycerol. If the knockout strain SGrmlB can anaerobically produce rhamnolipids using glycerol as carbon source, there is a new biosynthetic pathway of dTDP-l-rhamnose under anaerobic conditions (hypothesis 1). If not, the *rmlBDAC* genes control the anaerobic biosynthesis of dTDP-rhamnose, and the expression of *rmlBDAC* genes and other key genes are inhibited under anaerobic conditions (hypothesis 2). The transcriptomics results can provide the related information.

### Anaerobic production of rhamnolipids by strain SG and SGrmlB using glycerol

dTDP-Rhamnose and -hydroxy fatty acids are two required precursors for rhamnolipids biosynthesis. The *rmlBDAC* operon genes control the biosynthesis of dTDP-rhamnose, and -hydroxy fatty acids are derived from the de novo synthesis of fatty acids [19]. The de novo synthesis of fatty acids is central metabolic pathway in *P. aeruginosa*. Therefore, anaerobic synthesis of dTDP-rhamnose is one key metabolic pathway for rhamnolipid biosynthesis under anaerobic conditions.

**Fig. 3:**
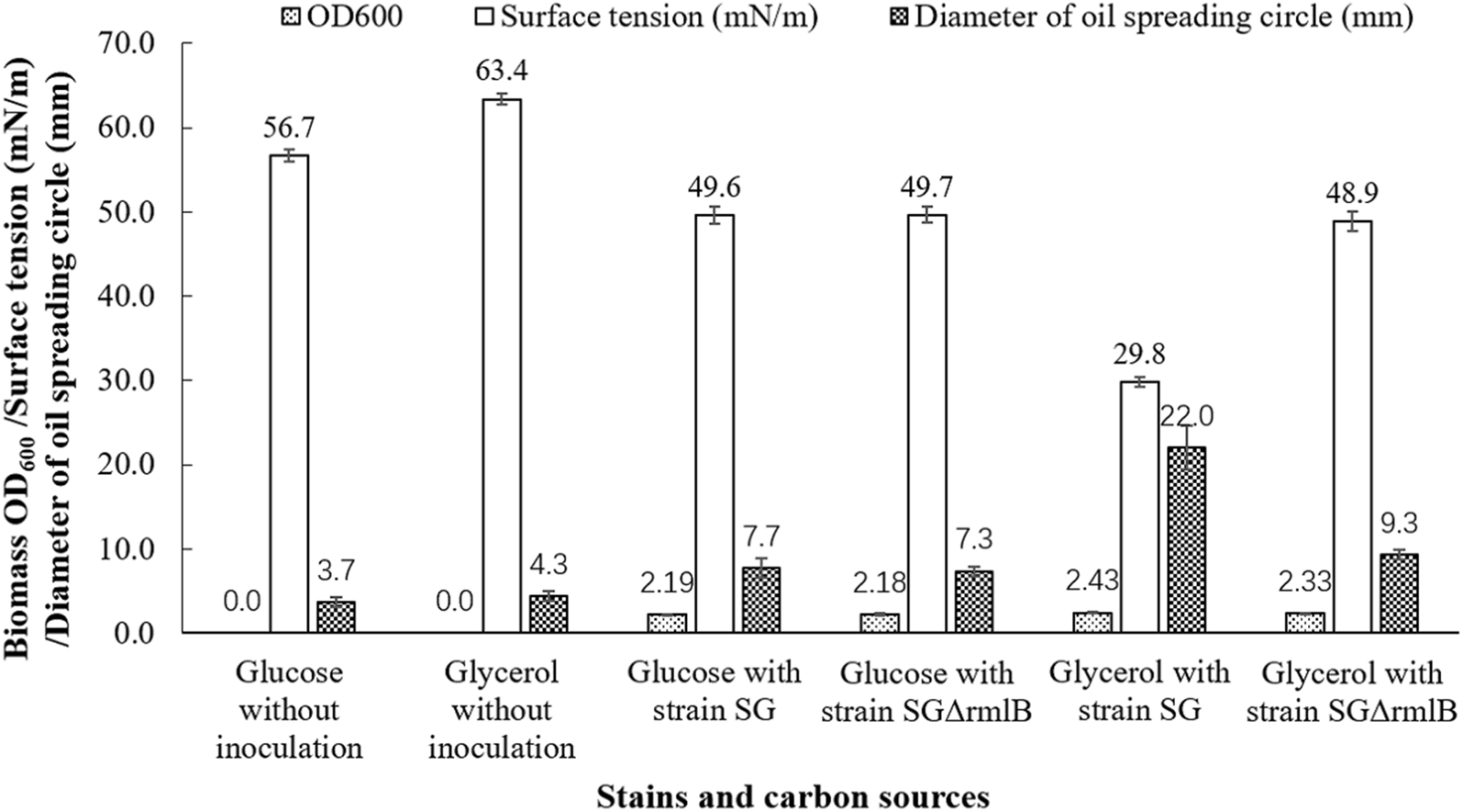
Anaerobic growth and production of rhamnolipids by the wild-type strain *P. aeruginosa* SG and the knockout strain *P. aeruginosa* SGrmlB using glucose and glycerol as carbon sources

The anaerobic cell growth and anaerobic rhamnolipids production of wild-type strain SG and the knockout strain SGrmlB were comparatively determined. If the knockout strain SGrmlB can anaerobically produce rhamnolipids using glycerol as carbon source, there is a new biosynthetic pathway of dTDP-l-rhamnose for rhamnolipids synthesis using glycerol under anaerobic conditions. If not, the *rmlBDAC* operon genes control the anaerobic biosynthesis of dTDP-rhamnose then for rhamnolipids synthesis using glycerol under anaerobic conditions. As shown in Fig.3, the knockout strain SGrmlB can anaerobically grow using both glucose and glycerol as carbon sources with OD_600_=2.18 and 2.33. The wild-type strain SG can anaerobically produce rhamnolipids using glycerol as carbon source, reducing surface tension to 29.8mN/m and forming oil spreading circle with diameter of 22.0mm. Although glycerol used as carbon source, the knockout strain SGrmlB can not anaerobically produce rhamnolipids, reducing surface tension to 48.9mN/m and forming oil spreading circle with diameter of 9.3mm. Results indicated that the *rmlBDAC* operon genes involved in the anaerobic biosynthesis of rhamnolipids using glycerol.

### Basic transcriptome results of strain SG using glycerol or glucose under anaerobic conditions

To understand why glycerol can be used for anaerobically synthesizing rhamnolipids by strain SG while glucose cannot, RNA sequencing (RNA-seq) was performed on strain SG using glucose and glycerol as carbon source under anaerobic conditions, respectively. The RNA-seq data were shown in Table 1. A total of 90270732 Raw reads were obtained. After quality control, 84703416 Clean Reads were obtained. Totally, 5875 genes were mapped to the reference genome of *P. aeruginosa* PAO1 (NC_002516). The RNA sequencing data have been deposited in NCBIs Sequence Read Archive (SRA) with Accession Number PRJNA670563 (https://dataview.ncbi.nlm.nih.gov/object/PRJNA670563?reviewer=q57rp98cvqi8nvid6dv8urgint). FPKM values of the mapped genes were calculated for analysis of the gene expression level. The FPKM value of one was used as the threshold to judge whether the gene was expressed or not. The correlation of gene expression levels between samples is an important indicator to test the reliability of experiments and the reasonableness of sample selection [31]. The closer the Pearson correlation coefficient squared (R^2^) is to 1, the higher the similarity of expression patterns between samples is and the less different genes among samples. In this study, Pearson correlation coefficient squared (R^2^) between samples in GluAn and samples in GlyAn were all greater than 0.92, which indicated the ideal sampling and experimental conditions.

**Table 1:** RNA-seq data of strain SG using glycerol and glucose as carbon sources under anaerobic conditions

Sample name	Raw reads	Clean reads	Clean bases (Gb)	GC content (%)	Total mapped reads	Mapped percentage (%)
GluAn-1	13878806	12993448	1.94	63.60	12542479	96.53
GluAn-2	16251408	15319168	2.30	63.86	14794181	96.57
GluAn-3	14265600	13323478	2.00	63.58	12748319	95.68
GlyAn-1	16223066	15051700	2.26	64.14	14573577	96.82
GlyAn-2	14887764	13860002	2.08	63.94	13433828	96.93
GlyAn-3	14764088	14155620	2.12	63.94	13733081	97.02

**Fig. 4:**
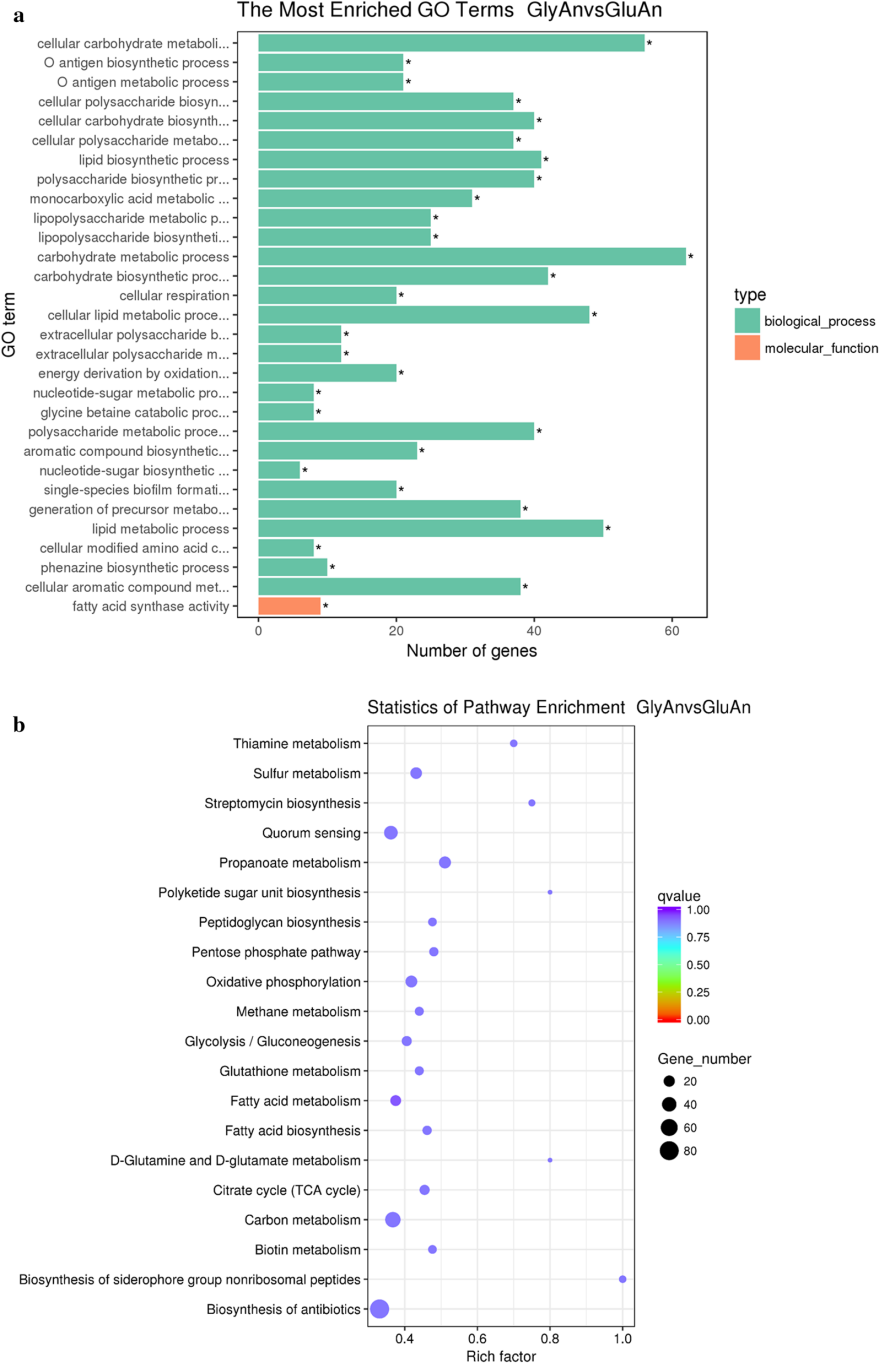
GO annotation and KEGG pathways enrichment of the differentially expressed genes between using GlyAn and GluAn **a** the most enriched GO Terms, the asterisk indicated that the GO item was significantly enriched (*P*<*0.05*); and **b** the statistics of KEGG pathways enrichment

According to the readCount data obtained from gene expression level analysis, a total of 972 significantly differentially expressed genes with log2(FoldChange) value>1 and q-value<0.05 were identified between strain *P. aeruginosa* SG using glycerol and glucose as carbon sources under anaerobic conditions. There were 549 up regulated genes and 423 down regulated genes in GlyAn samples vs GluAn samples. As shown in Fig.4a, the up regulated genes were significantly enriched into 37 GO terms belonging to two major functional categrories, molecular function and biological process. KEGG pathways enrichment analysis showed that the up regulated genes were enriched into 20 pathways (Fig.4b). Some of the enriched metabolic pathways are closely related to the biosynthesis of rhamnolipids, such as fatty acid biosynthesis and metabolism, quorum sensing, gluconeogenesis.

### Differentially expressed genes involved in anaerobic biosynthesis of rhamnolipids

As listed in Table 2, the genes related to the biosynthesis of dTDP-l-rhamnose and -hydroxy fatty acids, and the rhamnosyl transfer process pathways were picked out from the differentially expressed genes between GlyAn samples and GluAn samples. The precursor, dTDP-l-rhamnose, provides the glycosyl part for the biosynthesis of rhamnolipids. It can be initiated by the gluconeogenesis pathway or the EntnerDoudoroff pathway, and the *algC* gene and *rmlBDAC* operon genes control the dTDP-l-rhamnose biosynthesis [32, 33]. The another precursor, -hydroxy fatty acids, provides the lipid chain part for the biosynthesis of rhamnolipids. It can be initiated by the de novo synthesis of fatty acids. Fatty acid synthase system II, Ketolipoyl reductase (FabG) and peptide-transferase (RhlA) catalyze the biosynthesis of -hydroxy fatty acids [32, 33]. The two precursors, dTDP-l-rhamnose and -hydroxy fatty acids, were assembled to mono-rhamnolipids and di-rhamnolipids catalyzed by rhamnotransferase I (RhlB) and rhamnotransferase II (RhlC), respectively [34]. Rhamnotransferase I (RhlB) and rhamnotransferase II (RhlC) were coded by genes *rhlB* and *rhlC*, respectively. The genes *lasRI* and *rhlRI* in quorum-sensing pathways are responsible for activating the expression of genes, *rhlAB* and *rhlC* [34, 35]. Under anaerobic conditions, the related structural genes and regulatory genes, *rmlBDAC*, *rhlAB*, *rhlC*, *fabA*, *fabG* and *rhlRI*, *lasI*, were significantly up-regulated or activated when using glycerol as carbon source. Glycerol metabolism up-regulated the expression of genes in Quorum-sensing system, which promote the expression of genes *rmlBDAC*, *fabA*, *fabG*, *rhlAB* and *rhlC*. The anaerobic high expression of key genes is significant for *P. aeruginosa* producing rhamnolipids under anaerobic conditions.

**Table 2:** The differentially expressed genes related to the biosynthesis of dTDP-l-rhamnose and -hydroxy fatty acids, and the rhamnosyl transfer process

Gene id	Gene name	Readcount-GlyAn	Readcount-GluAn	log2(foldchange)	p-value	Gene description
PA5322	*algC*	3951.9	3245.6	0.28	0.43	phosphomannomutase AlgC
PA5161	*rmlB*	4139.6	1464.7	1.49	9.58E09	dTDP-d-glucose 4,6-dehydratase
PA5162	*rmlD*	1650.9	538.10	1.62	1.02E09	dTDP-4-dehydrorhamnose reductase
PA5163	*rmlA*	2270.2	789.6	1.52	8.69E09	glucose-1-phosphate thymidylyltransferase
PA5164	*rmlC*	2170.5	649.9	1.74	3.76E11	dTDP-4-dehydrorhamnose 3,5-epimerase
PA1609	*fabB*	2195.5	1304.6	0.75	1.65E03	beta-ketoacyl-ACP synthase I
PA1610	*fabA*	945.2	472.6	1.00	3.96E05	beta-hydroxydecanoyl-ACP dehydrase
PA2965	*fabF1*	1117.3	966.8	0.21	0.40	beta-ketoacyl-acyl carrier protein synthase II
PA1373	*fabF2*	109.6	395.1	1.85	2.57E12	3-oxoacyl-acyl carrier protein synthase II
PA2968	*fabD*	702.6	439.7	0.68	6.68E03	malonyl-CoA-[acyl-carrier-protein] transacylase
PA2967	*fabG*	2143.7	926.5	1.21	5.82E07	3-oxoacyl-[acyl-carrier-protein] reductase
PA3333	*fabH2*	677.1	10.0	6.08	2.65E70	3-oxoacyl-[acyl-carrier-protein] synthase III
PA3476	*rhlI*	5429.4	92.4	5.88	1.56E87	autoinducer synthesis protein RhlI
PA3477	*rhlR*	13664.1	732.3	4.22	1.93E56	transcriptional regulator RhlR
PA3478	*rhlB*	26462.6	243.7	6.76	1.28E91	rhamnosyltransferase chain B
PA3479	*rhlA*	42259.3	370.3	6.83	1.27E69	rhamnosyltransferase chain A
PA1130	*rhlC*	1418.9	16.1	6.46	2.61E86	rhamnosyltransferase 2
PA1432	*lasI*	1260. 4	196.1	2.68	1.95E23	autoinducer synthesis protein LasI

### Pathways and key genes for anaerobic synthesis of rhamnolipids using glycerol

In this study, the knockout strain SGrmlB could not anaerobically synthesize rhamnolipids even using glycerol as carbon source. Results showed that the pathways to metabolize glycerol to synthesize dTDP-l-rhamnose was also controlled by *rmlBDAC* operon genes under anaerobic conditions. The gene expression results showed that the related genes for rhamnolipids biosynthesis were highly expressed under anaerobic conditions using glycerol. The genes, *rmlBDAC*, *rhlAB*, *rhlC*, *fabA*, *fabG* and *rhlRI*, *lasI*, were significantly up-regulated expression using glycerol as carbon source, compared to using glucose under anaerobic conditions.

Based on the above results, Hypothesis 2 is more convincing (Fig.2). The anaerobic biosynthesis mechanism of rhamnolipids in *P. aeruginosa* was revealed at the gene level using glycerol as carbon source. The anaerobic biosynthetic pathway of rhamnolipids in *P. aeruginosa* SG were confirmed, involving the gluconeogenesis from glycerol, the biosynthesis of dTDP-l-rhamnose and -hydroxy fatty acids, and the rhamnosyl transfer process, which is consistent with the aerobic biosynthetic pathways in model strain *P. aeruginosa* PAO1. The key genes involved in anaerobic biosynthesis of rhamnolipids are *rmlBDAC*, *rhlABRI*, *rhlC*, *fabA*, *fabG* and *lasI*. Glycerol metabolism up-regulated the expression of genes in Quorum-sensing system (*rhlRI* and *lasI*), which promote the expression of genes *rmlBDAC*, *rhlAB* and *rhlC*, thus enable *P. aeruginosa* to anaerobically produce rhamnolipids using glycerol.

### Enhanced oil recovery by anaerobic production of rhamnolipids

After the first water flooding, 45.21% of the oil was recovered from the core model. At the end of the second water flooding, 54.88% of oil was recovered. In-situ anaerobic production of rhamnolipids by engineered strain *P. aeruginosa* PrhlAB enhanced 9.67% of oil recovery. In our previous study [13], in-situ anaerobic production of rhamnolipids by wild strain *P. aeruginosa* SG enhanced 8.33% of oil recovery. Increasing the copy number of *rhlAB* genes in *P. aeruginosa* SG, the engineered strain *P. aeruginosa* PrhlAB can produce more rhamnolipids under anaerobic conditions [15]. Improving the anaerobic production of rhamnolipids better enhanced oil recovery in core flooding test. Future research will be focused on further enhancing the anaerobic production yield based on the anaerobic biosynthetic mechanism of rhamnolipids.

### Other prospect of the anaerobic production of rhamnolipids

*P. aeruginosa* strains are facultative bacteria [10], but the vast majority of studies on the biosynthesis of rhamnolipids in *P. aeruginosa* were focused on aerobic biosynthesis [19, 34, 35]. Anaerobic biosynthesis of rhamnolipids is promising to the in-situ applications in anoxic environments, such as deep soil, oil reservoirs, sediments [4, 6, 13]. Rhamnolipids products have also been partially commercialized in some fields. Nevertheless, the necessary aeration and defoaming processes in the aerobic fermentation of rhamnolipids lead to high production costs, which affects the large-scale industrial application of rhamnolipids [12, 36]. The foam problem in production of rhamnolipids can be avoided by anaerobic fermentation without aeration. The low anaerobic production yield of rhamnolipids was the bottleneck for in-situ enhanced oil recovery, and the foamless fermentation. Enhancing the anaerobic production yield of rhamnolipids is of great importance. In the future, the revealed biosynthetic pathways and key genes involved in anaerobic biosynthesis of rhamnolipids would be regulated and modified to enhance *P. aeruginosa* to anaerobically produce more rhamnolipids. Besides, -hydroxy fatty acids are precursor substrates of rhamnolipids synthesis as well. Fatty acid synthesis requires a lot of reducing power (NADPH or NADH_2_). Compared with anaerobic conditions, aerobic conditions are conducive to cell synthesis of reducing power, which promotes fatty acid synthesis and ultimately enhances rhamnolipids synthesis. Increasing the reducing power level in *P. aeruginosa* cells will also enhance the production of rhamnolipids under anaerobic conditions. Results of this study would be significant for the metabolic engineering of *P. aeruginosa* to enhance anaerobic production of rhamnolipids.

## Conclusion

*P. aeruginosa* can anaerobically produce rhamnolipid using glycerol rather than glucose. Two possible hypotheses on anaerobic biosynthesis of rhamnolipid using glycerol were proposed. The *rmlBDAC* operon genes involved in the anaerobic biosynthesis of rhamnolipid using glycerol. The expression of key genes was inhibited under anaerobic conditions when *P. aeruginosa* using glucose. The highly anaerobic expression of key genes enables *P. aeruginosa* to anaerobically biosynthesize rhamnolipids using glycerol. The anaerobic biosynthetic pathway of rhamnolipid using glycerol was confirmed. Improving the anaerobic production of rhamnolipids better enhanced oil recovery. This study fills the gaps in the anaerobic biosynthesis mechanism of rhamnolipids.

## Data Availability

The datasets supporting the conclusions of this article are included within the article.
